# Long-term growth patterns of vestibular schwannomas after stereotactic radiotherapy: delayed re-growth

**DOI:** 10.1007/s00405-022-07281-2

**Published:** 2022-02-07

**Authors:** Owen Conlan, Georgios Kontorinis

**Affiliations:** 1 School of Medicine, University of Glasgow, Glasgow, UK; 2 Department of Otolaryngology-Head and Neck Surgery, Queen Elizabeth University Hospital, 1345 Govan Road, Glasgow, G51 4TF UK

**Keywords:** Growth, Radiotherapy, Treatment, Vestibular schwannoma

## Abstract

**Purpose:**

To determine the long-term outcomes of patients with vestibular schwannomas (VS) after stereotactic radiosurgery (SRS) who experience delayed tumour regrowth.

**Methods:**

We carried out a retrospective case series in tertiary university settings. We included patients with VS with initial response to SRS and delayed regrowth, assessing a database of 735 patients with VS and 159 patients who had SRS as sole treatment. Following SRS, all patients had clinical follow-up and serial magnetic resonance imaging (MRI). We documented the post-SRS clinical assessment, pre- and post-SRS VS size as per MRI in predetermined time periods, response to treatment and rate of (re-) growth and the final outcome in each case.

**Results:**

We identified six patients with good initial response but delayed VS regrowth at a faster rate than pre-SRS. The mean growth rate for these VS was 0.347 mm/month (range 0.04–0.78 mm/month) prior to treatment; the mean growth rate at the time of delayed re-growth was 0.48 mm/month (range 0.17–0.75 mm/month); this did not reach the level of statistical significance (*p* = 0.08). This regrowth occurred at a mean time of 42 months (range 36–66 months) post-SRS and stopped 22 months (mean, range 12–36 months) post regrowth detection in all cases.

**Conclusions:**

Given that delayed post-SRS VS regrowth can occur in approximately 4% of the treated cases, it is important to continue close clinical and radiological follow-up. Despite this abnormal behaviour, VS do stop growing again; still, patients should be made aware of the possibility of this uncommon VS behaviour following SRS.

## Introduction

Vestibular Schwannomas (VS) are benign tumours arising from Schwann cells of the eighth cranial nerve. The prevalence of these is thought to be roughly 2 in 10,000 [1]. The primary diagnostic imaging tool is Magnetic Resonance Imaging (MRI), and is also used in the surveillance of small VS [2, 3]. In larger or growing VS, microsurgical resection or Stereotactic Radiosurgery (SRS), delivering high-dose radiation to a precise location, may be required [4–9].

SRS has been shown to provide a very good tumour control rate of > 90% [9–11] with a relatively low morbidity in the treatment of VS. Following SRS, patients are typically closely monitored clinically and with MRI. This is done to monitor tumour size and identify stability or regression of the tumour. Although it is widely believed that VS that have responded well to SRS may not need any long-term follow-up, recent work has reported on delayed failure of SRS with VS that responded to the treatment but later re-grew [12].

Despite our better understanding of VS natural history, further reports on delayed SRS failures and the long-term outcomes of those cases are missing from the literature. Our aim was to analyse and determine the long-term outcomes of patients with VS with good initial response to SRS (regression) but delayed regrowth; as to our knowledge, such data are not available, until now.

## Methods

### Basic settings

A retrospective case series was carried out in a tertiary university centre with a catchment area of approximately 2.2 million people. Due to its retrospective, anonymised setup, the study was considered as an audit and no further ethical approval by the Research Ethical Committee was required; Caldicott guardianship was granted for this study.

### Patient selection

Patients were identified through the regional database of patients with a VS covering a 20-year period (2000–2019); this dataset included complete data for 735 patients with VS with a total of 163 being treated with SRS; nine of these patients were treated with surgery and SRS, either as a consequence of failure of SRS (five patients) or due to significant growth of residual tumour following resection (four patients). We finally included 159 patients (163 minus the four patients who had primary surgery) treated with solely primary SRS.

This set was then filtered down by identifying those who responded to SRS based on their post-SRS imaging. Finally, we identified only patients whose VS had an initial period of tumour regression or stability post-SRS then experienced regrowth; this was performed through robust retrospective review of serial imaging. The inclusion criteria for these patients were as follows:

- Patients who experienced delayed regrowth of the VS following good initial response to SRS
- Patients with detailed imaging including at least two MRI scans prior to SRS to determine pre-treatment growth, post-SRS scans showing definite regression, MRI scans showing regrowth of the VS following that regression period and follow-up of at least two years following the regrowth
- Patients who had a T1 weighted MRI both pre and post delayed growth
- Patients who had SRS as sole treatment method

Patients who had VS due to Neurofibromatosis Type 2 were excluded due to the nature of the disease.

All included patients were treated with SRS in the same centre receiving 12–13 Gy, single shot treatment.

### Imaging and follow-up

All included patients have had serial 1.5 T MRI of the internal auditory meatus including steady-state MRI sequences, pre- and post-contrast (gadolinium) T1 and T2 weighted sequences with reconstruction at least on the axial and coronal plane. Due to the long time period of the study, the patients were scanned in different scanners.

All patients were scanned based on our local protocol and predetermined time intervals, unless otherwise clinically indicated (first scan 12 months post-SRS, then annually for the first 5 years, additional scan year 7 and 10 following SRS).

We measured the maximum intracranial diameter (cerebello-pontine angle) either on the axial or coronal plane in mm. Due to the imaging limitations, we defined as growth or regression a change of at least 1.5 mm in linear measurements between scans; this was based on the accuracy and resolution-limitations of the used MRI scanners.

In this cohort no VS were located entirely intracanalicularly, with all extending into the cerebello-pontine angle.

### Examined factors and analysis

From the selected patients, we recorded age and sex, as well as the size of the tumour and the growth rate both prior to and after treatment. We also recorded the total and post-SRS/ regrowth follow-up. The composition of the tumour, whether it was solid, cystic or both was also recorded in addition to any cystic degeneration following SRS. Due to the long period covered by the study and software issues when analysing past scans, we were not able to include volumetric measurements. Therefore, we used the growth rate as an additional measurement. For the identified cases with delayed re-growth the presented measurements are based on the scan report and additional confirmation by one of our Neuroradiologists; although we were prepared to use consensus for any discrepancies, this was not required. Of note, as regular practice and standard of care in our Institution, all scans are being reviewed in a regular multidisciplinary environment to ensure accurate measurements and management.

We also recorded the growth or regression as well as the growing rate in mm/month as per previous works by Fayad et al. [13] and Ton et al. [14]. We recorded the growing rate from the time of diagnosis to the time of SRS (period 1, indicating period of initial growth), from the time of SRS to the smallest diameter (period 2, indicating response to treatment) and from the time of regrowth till the end of the follow-up (period 3, indicating re-growth).

Patient symptoms were not considered due to the long period of time that our study covered as well as its retrospective nature. As at the time of re-growth all patients had non-serviceable hearing, we did not include detailed hearing assessment data. However, we included any clinical changes at the time of the re-growth.

We organised all data onto an Excel Spreadsheet (Microsoft) and used the Mann Whitney test to compare the growth rates between the pre-treatment and post-regrowth rates; the level of significance was set at 0.05.

## Results

### Tumour growth pre to post SRS

Out of the 163 patients treated with SRS, in 159, this was the primary treatment with tumour control in 154 patients (96.8%) at the time of this study. Out of these 154 patients, we identified six patients (3.8% of the whole SRS cohort and 3.9% of the tumour control cohort) with delayed VS regrowth post-SRS that fulfilled the inclusion criteria (Table 1). Two of the six VS in this study also had a cystic component. No further changes within the VS such as central necrosis, were identified.

**Table 1:** Basic demographics and size of the VS

Patient	Age (years)	Gender	Initial size (mm)	Pre-treatment size (mm)	Post-treatment size (mm)	Regrowth size (mm)
1	58	Female	11	14	11.5	16
2	59	Female	16	17	14	16
3	46	Male	1	15	15	19
4	71	Female	7	15	9.5	32
5	64	Male	1	14	10	19
6	65	Female	6	13	18.5	21

Size refers to the maximum intracranial diameter (linear measurements of the length of tumour extending beyond the porous); all sizes refer to the final size for each time period, just before SRS, just before starting re-growing and final size

The mean initial size of the tumour was 7 mm (range 1–16 mm) with a mean follow-up until receiving SRS being 24 months (range 12–36 months). The mean growth rate for these VS was 0.347 mm/month (range 0.04–0.78 mm/month) with a mean size at treatment 14.67 mm (range 13–17 mm). The mean growth rate after receiving SRS was − 0.09 mm/month (range − 0.18–0 mm/month) (Table 2).

**Table 2:** Growth rates for each period

Patient	Growth rate till SRS (mm/month)*	Growth rate from SRS to regrowth (mm/month)	Growth rate at regrowth (mm/month) *
1	0.125	− 0.07	0.375
2	0.08	− 0.08	0.17
3	0.78	0	0.125
4	0.04/0.58	− 0.18	1.25
5	0.36	− 0.1	0.75
6	0.39	0.1/0	0.2

*p = 0.08

The mean reached smallest tumour size following SRS was 13.08 mm (range 10–18.5 mm).

### Tumour growth post SRS to end of regrowth

**Fig. 1:**
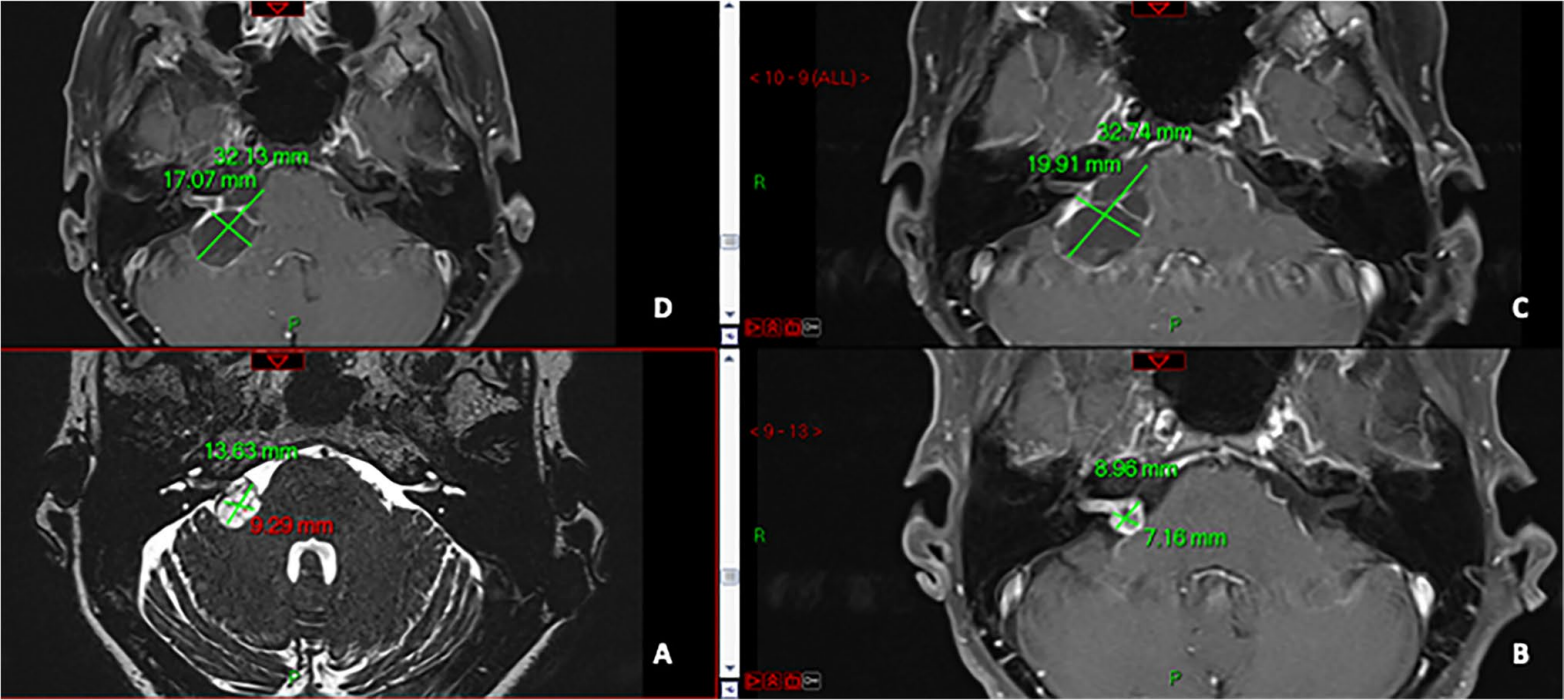
The changes in the size of a right VS in an elderly patient in axial 1.5 T MRI (anticlockwise): **A** steady state MRI prior to treatment showing the right VS with 13.6 mm maximum intracranial diameter, with good response (post-gadolinium T1-weighted) following the treatment at just under 9 mm diameter (**B**); cystic degeneration 30 months post-SRS with significant enlargement and temporal balance deterioration with just under 33 mm maximum intracranial diameter (**C**) and stable appearances (with a small decrease in size) in an 18-month follow-up following the re-growth at 32 mm (**D**)

**Fig. 2:**
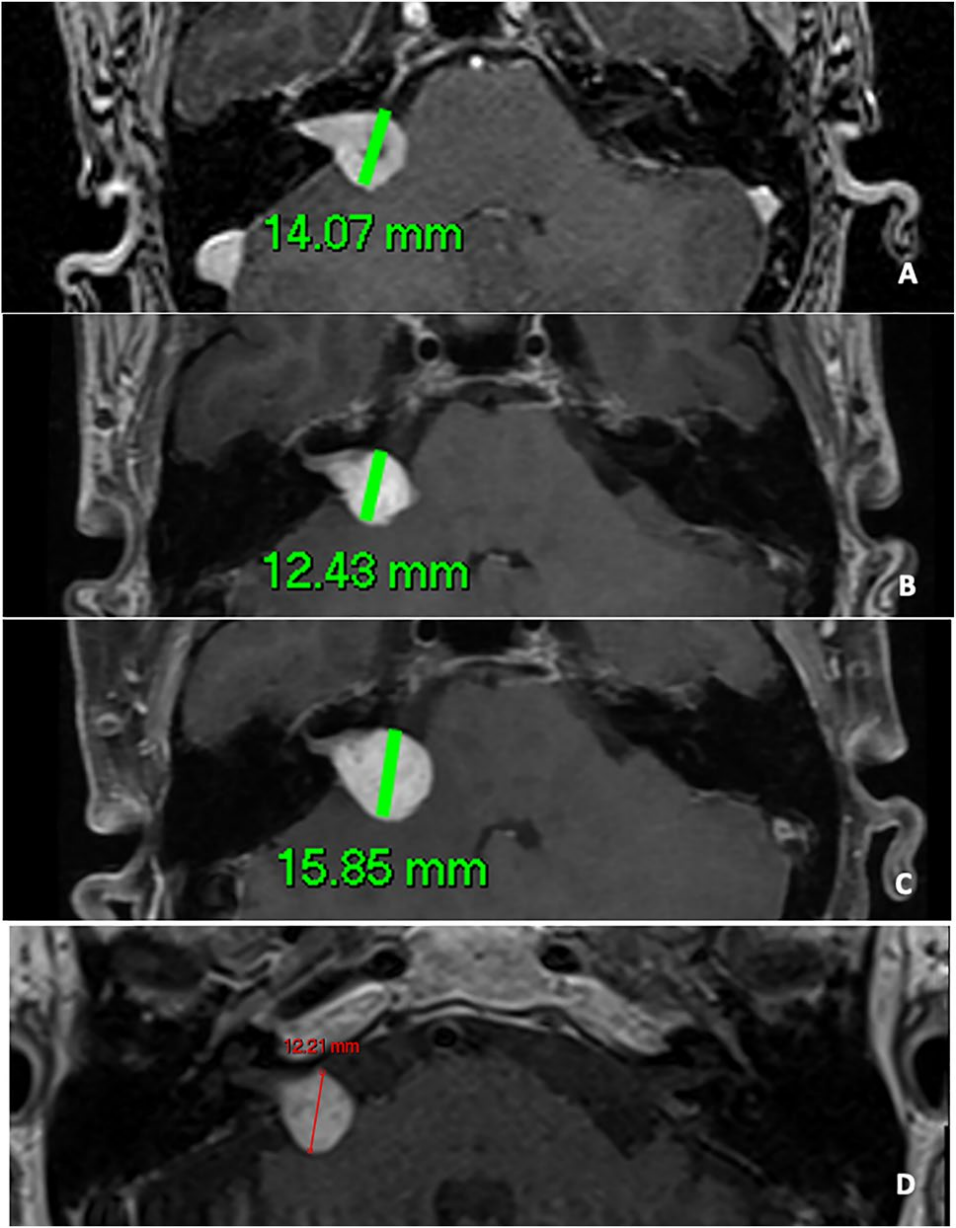
Female patient with right VS previously presented by Stapleton et al. [12] (**A** shows the pre-treatment size, **B** the post-treatment and **C** the post-SRS growth) with long-term follow-up (36 months following the re-growth) showing decrease in size

**Fig. 3:**
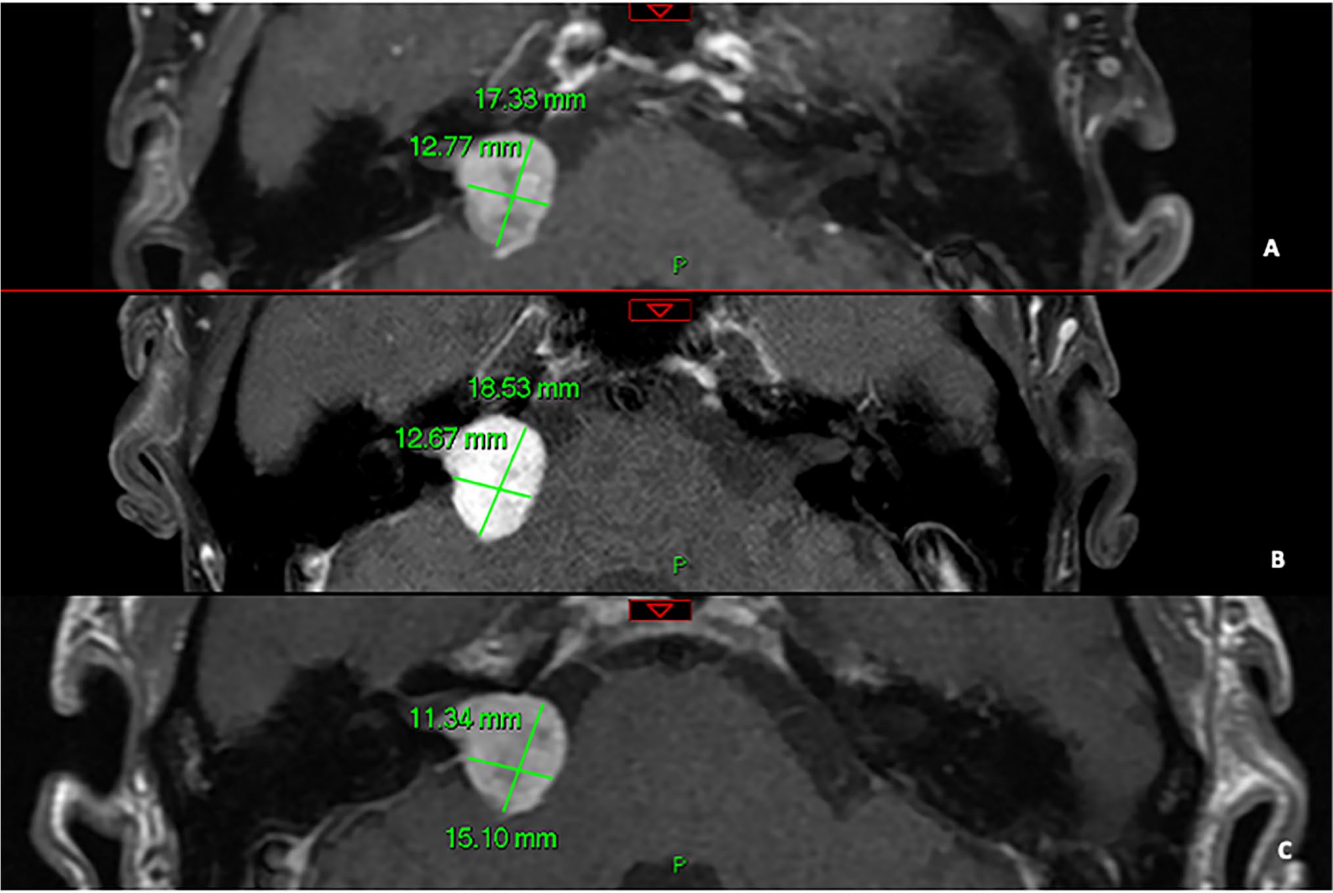
Post-SRS axial, T1-weighted post-gadolinium 1.5 T in a male patient with right VS (**A**) showing the increase in size 6 years post-treatment (**B**) and further stabilisation nearly 2 years later (**C**)

The mean time following SRS when regrowth began was 42 months after SRS (range 36–66 months), with a mean growth rate of 0.48 mm/month (range 0.17–0.75 mm/month); this growing rate was higher than the pre-SRS growth rate but did not reach the level of statistical significance (*p* = 0.8) (Table 2). The mean final VS size was 20.5 mm (range 16–32 mm) with all VS stabilising within their follow-up period (mean: 22 months post-regrowth, range: 12–36 months). Figures 1,  2 and 3 demonstrate the changes in size in some of our patients.

No clinical features, including trigeminal or facial nerve symptoms, were linked to these changes apart from temporary balance deterioration in one patient, which resolved within 3 months spontaneously; the resolution of the balance symptoms was not linked chronologically to tumour re-growth arrest.

## Discussion

### Main findings

Despite our more advanced understanding of VS natural history, delayed regrowth after receiving SRS is not well documented in the literature, nor is the long-term outcomes of those cases. Previously, we have reported on the short-term outcomes of delayed regrowth following SRS in VS [12]; herein we extend our cohort and add long-term data to significantly improve our understanding. Delayed regrowth can be seen in just under 4% of VS treated with SRS. Despite this new growth at a faster (but not statistically significant) rate than that of prior to receiving SRS, the VS do stop growing again, and therefore, no additional intervention is required.

However, this does demonstrate that long-term monitoring of VS is required, even in those that show a good response over a long period. It was also observed that a cystic component of the VS was not necessarily related to growth.

We do recognise that the post-SRS growing patterns have been well examined in the literature [7–12, 15], showing that post-SRS growth is not uncommon and not, usually, of major concern. Indeed, previous study has shown 14% of VS showing enlargement following SRS [15], with most of these schwannomas not needing any further treatment; such initial growth has been linked to post-SRS inflammation/ oedema [7–11]. However, here we show good initial response and delayed re-growth, which introduces a completely different concept. Such delayed re-growth introduces a few challenges that are discussed below: delayed but real treatment failure, the challenge of dealing with the patients’ anxiety following such VS behaviour but also the possibility of malignant transformation following SRS.

### Clinical implications and long-term imaging

The growing patterns of VS can still be unpredictable, even after receiving SRS. There is the possibility of malignant transformation following SRS [16] although we did not have such a case within our cohort. This is an important factor to consider in patients with VS that demonstrate such bizarre behaviour post-SRS. Overall, the number of reported cases of malignant transformation following SRS is very low; additionally, one could argue that as these tumours had not been biopsied, they were not VS in the first place. Thus, while such a possibility should be considered, it appears extremely low and would not warrant immediate intervention, unless there are concerning radiological or even clinical features suggesting this possibility.

As above, that close imaging and clinical assessment is required, it is also important to carefully consult with patients as to give neither false alarms nor false reassurance. There is a difference between post-SRS growth, which is well recognised and reported [7–12, 15] and delayed re-growth following good response that is described here. This is not the usual VS behaviour; it can cause initial concerns to the treating doctor and the patient and will need to be recognised and thoroughly explained to the patient. While none of the cases needed additional treatment, it needs to be highlighted to the patient to avoid unnecessary anxiety and concerns. In addition, patients should be made aware of such VS behaviour prior to any SRS treatment, as this could affect decision making.

It is worth mentioning that while in a previous report this delayed regrowth raised concerns, our current study with long-term follow-up and a larger cohort indicating later stabilization of the VS provides additional reassurance and evidence. Still, it is sensible to keep VS with such ‘abnormal’ behaviour under close surveillance until they stop growing again.

Overall, there are no known evidence-based, globally agreed guidelines on the optimal post-SRS monitoring period. Empirically, as per the local (and national practice) a 10-year period is recommended. Based on the presented data, we have not identified any re-growth after the 6-year mark; thus, the 10-year period is reasonable, justifiable and supported by the presented data.

### Strengths and limitations of this study

The main limitations to this study are the small cohort size and the retrospective nature and its associated bias. In order to overcome such limitations, we used standardised and more detailed measurements, such as growth in mm/month, which allowed us a more accurate and thorough way of quantifying growth. While one could argue the absence of volumetric measurements as an additional weakness (this was not possible due to software issues analysing past scans), we used linear measurement limitations, based on the used scanners but also added a second measurement (growth rate in mm/ month) to better address the growing patterns through the long covered period. Additionally, all scans and measurements have been processed and confirmed in a multidisciplinary environment, ensuring accuracy.

Additionally, given the rarity of VS and limited examined group (ones treated solely with SRS), our initial SRS cohort of 159 patients is small but clinically significant. Given the lack of similar studies in the literature, this work provides us with the first indication that long-term monitoring is required, although further data and an increased cohort size would strengthen our findings. As above, our findings would support the experience-based monitoring of 10 years post-SRS.

*In conclusion*, delayed SRS regrowth following good initial response can occur in approximately 4% of the treated patients. Despite these initial concerns, VS do stop growing again and remained static for the duration of this study. Patients should be aware of this uncommon ‘abnormal’ VS behaviour following SRS, which does not raise long-term concerns.

## Data Availability

Not applicable.
